# GM-CSF: A Double-Edged Sword in Cancer Immunotherapy

**DOI:** 10.3389/fimmu.2022.901277

**Published:** 2022-07-05

**Authors:** Anil Kumar, Adeleh Taghi Khani, Ashly Sanchez Ortiz, Srividya Swaminathan

**Affiliations:** ^1^ Department of Systems Biology, Beckman Research Institute of City of Hope, Monrovia, CA, United States; ^2^ Department of Hematological Malignancies, Beckman Research Institute of City of Hope, Monrovia, CA, United States

**Keywords:** GM-CSF (granulocyte-macrophage colony-stimulating factor), tumor immune microenvironment, cancer treatment, anti-tumor cytokines, pro-tumor cytokines

## Abstract

Granulocyte-macrophage colony-stimulating factor (GM-CSF) is a cytokine that drives the generation of myeloid cell subsets including neutrophils, monocytes, macrophages, and dendritic cells in response to stress, infections, and cancers. By modulating the functions of innate immune cells that serve as a bridge to activate adaptive immune responses, GM-CSF globally impacts host immune surveillance under pathologic conditions. As with other soluble mediators of immunity, too much or too little GM-CSF has been found to promote cancer aggressiveness. While too little GM-CSF prevents the appropriate production of innate immune cells and subsequent activation of adaptive anti-cancer immune responses, too much of GM-CSF can exhaust immune cells and promote cancer growth. The consequences of GM-CSF signaling in cancer progression are a function of the levels of GM-CSF, the cancer type, and the tumor microenvironment. In this review, we first discuss the secretion of GM-CSF, signaling downstream of the GM-CSF receptor, and GM-CSF’s role in modulating myeloid cell homeostasis. We then outline GM-CSF’s anti-tumorigenic and pro-tumorigenic effects both on the malignant cells and on the non-malignant immune and other cells in the tumor microenvironment. We provide examples of current clinical and preclinical strategies that harness GM-CSF’s anti-cancer potential while minimizing its deleterious effects. We describe the challenges in achieving the Goldilocks effect during administration of GM-CSF-based therapies to patients with cancer. Finally, we provide insights into how technologies that map the immune microenvironment spatially and temporally may be leveraged to intelligently harness GM-CSF for treatment of malignancies.

## 1 Role of GM-CSF in Homeostasis of Immune Cells

### 1.1 GM-CSF: Production, Receptors, and Signaling

Granulocyte-macrophage colony-stimulating factor (GM-CSF) is a glycoprotein best known for its role in myelopoiesis and myeloid cell function (1, 2). As the name indicates, GM-CSF promotes the generation of polymorphonuclear neutrophils (PMNs, a type of granulocyte), monocytes, macrophages, and dendritic cells (DCs) from hematopoietic progenitor cells (HPCs) in the bone marrow (1, 3–6). GM-CSF was first isolated from LPS-treated mouse lung-conditioned medium (3). Surprisingly, GM-CSF−/− mice are healthy and fertile with normal basal hematopoiesis; however, these mice develop lung abnormalities (7).

Although primarily associated with the stimulation of cells in the myeloid lineage, GM-CSF also regulates the activity of non-hematopoietic cells including epithelial cells, vascular endothelial cells, and fibroblasts (8). For example, GM-CSF is involved in epithelial cell proliferation and plays an important role in the maintenance and repair of the intestinal mucosal lining (9). GM-CSF also stimulates vascular endothelial cells and fibroblasts to modulate inflammation and autoimmunity (10).

GM-CSF is produced by lymphocytes, macrophages, fibroblasts, endothelial cells, chondrocytes, and tumor cells in response to immunogenic stimuli such as cytokines and toll-like receptor (TLR) agonists (11, 12). Cytokines such as interleukin (IL)-10, IL-4 and interferon (IFN)-γ, the immunosuppressive drug Cyclosporin A, and glucocorticoids inhibit the production of GM-CSF (13). Despite being one of the major sources of GM-CSF, T-cells lack the GM-CSF receptor (GM-CSFR) (12). However, poor T-cell responses to antigenic challenge in GM-CSF−/− mice (14) suggest that GM-CSF indirectly regulates T-cell mediated immunity.

**Figure 1:**
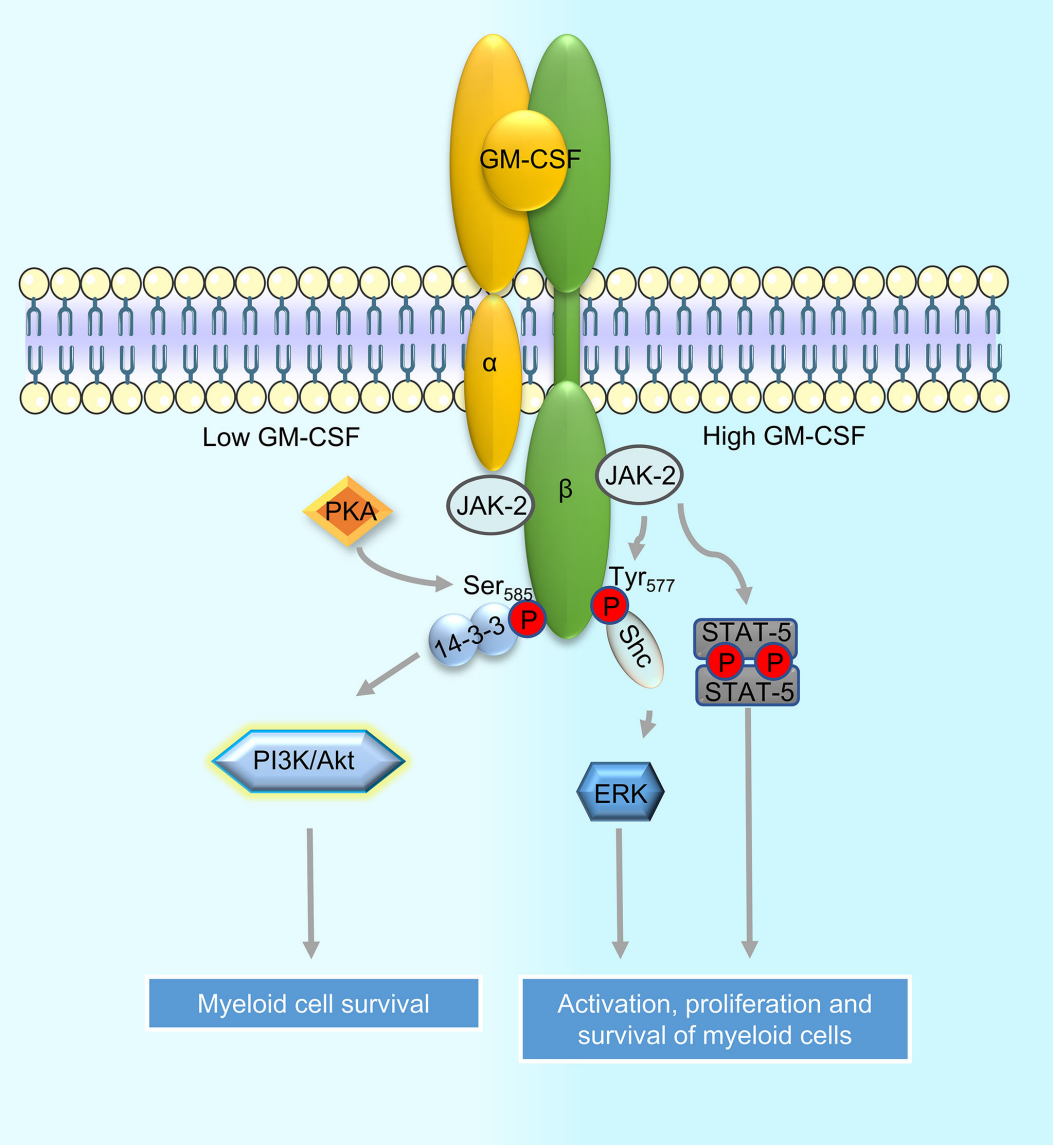
Signaling downstream of the GM-CSF receptor in myeloid cells. Binding of GM-CSF to the alpha chain of the GM-CSF receptor (GM-CSFR) leads to its dimerization with the signaling beta chain subunit. Beta chain-associated JAK2 then promotes receptor transphosphorylation and initiates downstream signaling. Depending on the sites of phosphorylation by protein kinases on the beta chain, specific adaptors are recruited to activate downstream signaling cascades such as the PI3K and MAPK pathways; recruitment of adapter protein 14-3-3 to phosphorylated Ser585 on beta chain leads to activation of the PI3K signaling while recruitment of Shc to phosphorylated Tyr577 leads to the activation of MAPK/ERK signaling. JAK2 bound to GM-CSFR can also directly activate STAT5 phosphorylation. Activation of PI3K downstream of GM-CSFR leads to myeloid cell survival whereas activation of MAPK/ERK and STAT5 induce proliferation of cells in addition to promoting their survival.

GM-CSFR is composed of one alpha chain and one signaling beta chain subunit (15, 16). The beta chain is shared with IL-3 and IL-5 receptors (17). GM-CSFR is expressed on myeloid cells (18), B cells (19), and non-hematopoietic cells including brain cells (neurons, astrocytes, and ependymal cells) (19), endothelial and alveolar cells (12). Activation of the GM-CSF receptor is identical to other class I cytokine receptors and requires receptor dimerization and tyrosine transphosphorylation of its cytoplasmic domains. GM-CSFR does not have innate tyrosine kinase activity, so it associates with the Janus Kinase 2 (JAK2) tyrosine kinase. JAK2 is needed for βc transphosphorylation and the initiation of signaling. The cytoplasmic domains of both GMRα and βc are essential for receptor activation but only βc associates with JAK2 (20–22). The activation of JAK2 initiates phosphorylation and dimerization of the signal transducer and activator of transcription 5 (STAT5). STAT5 dimers then migrate to the nucleus and initiate the transcription of genes such as PIM1 and cytokine-inducible SH2-containing protein (CIS) resulting in the differentiation of the target cell (10, 23). GM-CSFR activation and consequently JAK2 phosphorylation has also been shown to activate other intracellular signaling pathways such as those driven by the phosphatidylinositol 3 kinase (PI3K) and mitogen-activated protein kinases (MAPK). The pleiotropic functions of GM-CSF are regulated by the mutually exclusive phosphorylation of βc at Ser585 (low concentration) by protein kinase A (PKA) and at Tyr577 (high concentration) by tyrosine kinases (24). Phosphorylation of βc at Ser585 leads to the recruitment of the adaptor protein 14-3-3, a dimeric protein whose association with βc is required for PI3K recruitment and activation of cell survival signals (25). On the other hand, phosphorylation of βc at Tyr577 induces the recruitment of the adaptor protein Shc and phosphorylation of JAK2/STAT5 and extracellular signal-regulated kinase (ERK) ultimately resulting in the activation, proliferation, and survival of the target cell (24). The signaling cascades activated downstream of the GM-CSFR are described in **Figure 1**.

### 1.2 Role of GM-CSF in Myelopoiesis

Myelopoiesis is the differentiation of cells into the myeloid, non-lymphoid cell lineage. Commitment to myeloid lineage is triggered by binding of GM-CSF to GM-CSFR on myeloid cell precursors, followed by a cascade of signaling events downstream of the GM-CSFR (**Figure 1**) that ultimately lead to the production of myeloid-specific transcription factors including PU.1 and interferon regulatory factor 4 (IRF4) (1, 26). The importance of GM-CSF in cells of the myeloid lineage has been shown in GM-CSF-transgenic (Tg) mice where counts of myeloid subsets including macrophages, neutrophils, and eosinophils are substantially increased in comparison to control (27, 28). Studies in GMCSF−/− mice show that GM-CSF drives emergency myelopoiesis in response to infection, cancer, and stress but is dispensable for basal myelopoiesis (29, 30). However, some recent reports suggest that GM-CSF is required for some specialized aspect of basal hematopoiesis including the production of alveolar macrophages and dermal and lamina propria DCs (called migratory DC) (31, 32).

#### 1.2.1 GM-CSF Promotes the Generation of Monocytes

Monocytes, which constitute 2-8% of WBCs, are precursors to macrophages and dendritic cells (DCs). During normal hematopoiesis, CD14^+^ monocytes develop in the bone marrow from the common myeloid progenitor (CMP) with support from GM-CSF (33). Monocytes then home to the blood. Monocyte production is enhanced under conditions of inflammation, tumors, and chronic stress (34). Emergency myelopoiesis under inflammatory conditions is triggered by increased secretion of GM-CSF and IFN-γ by activated CD4^+^ T-helper cells (35). This drives macrophage/DC progenitors (MDPs) and common monocyte precursors (cMoPs) into the cell cycle. Increased amounts of classical monocytes are then released into the blood (35). Antibody-mediated blockade of GM-CSF converts the inflammatory profile of monocytes to an immunomodulatory one by reducing human leukocyte antigen (HLA)-DR, CD86, IL-1β and tumor necrosis factor (TNF)-α and by increasing their IL-10 production (36). Despite not impacting basal myelopoiesis (29), GM-CSF induces the differentiation of alveolar macrophages as exemplified by lymphoid hyperplasia and perturbations in pulmonary homeostasis in the absence of GM-CSF (37–39).

#### 1.2.2 GM-CSF Promotes the Generation of PMNs

PMNs, also called granulocytes, are a type of WBC which contain granules with enzymes that are released during infections and allergic reactions. PMNs include neutrophils, eosinophils, and basophils and form 45-75% of WBC. Of these, neutrophil is a key innate immune subset that protects against bacterial, viral, and fungal infections. GM-CSF directly and synergistically with other hematopoietic growth factors (G-CSF and IL-6) (40, 41), stimulates the proliferation of neutrophils from CMP, as demonstrated by *in vitro* and *in vivo* studies using overexpression of GM-CSF, injection of recombinant GM-CSF, and GM-CSF deficient mice (11, 27, 29, 42). GM-CSF−/− mice are unable to control listeria monocytogenes infection because of decreased neutrophil infiltration into the peritoneal cavity which is the site of infection (29).

#### 1.2.3 GM-CSF Promotes the Generation of DCs

Dendritic cells are antigen-presenting cells that link innate and adaptive immunity. DCs, which form 1% of WBCs are specialized in antigen capture, processing, and presentation, and bridge innate and adaptive immune responses. GM-CSF supports development of the common DC progenitor from HSCs (5, 43, 44) as well as differentiation of DCs from monocytes (45). Mice injected with GM-CSF and *GM-CSF-Tg* mice harbor substantially increased numbers of splenic and thymic DCs in comparison to their normal counterparts, thus corroborating that GM-CSF promotes *in vivo* expansion of DCs (5, 6, 43, 44, 46). Among DC subsets, GM-CSF drives the genesis of dermal DCs and lamina propria non-lymphoid tissue DCs (47, 48), but hardly influences lymphoid tissue resident DCs (46, 49). However, more recently, GM-CSF has been shown to promote the terminal differentiation of committed plasmacytoid DC (pDCs) (50). GM-CSF regulates the differentiation of DCs through JAK2-STAT5 and MEK/ERK signaling. On the other hand, PI3K-PKB signaling promotes expansion and survival of DC precursors but not their differentiation, downstream of GM-CSF (26).

## 2 GM-CSF Drives Both Tumor Suppression and Tumor Progression

By exerting its effects on a wide range of immune and non-immune cells, GM-CSF modulates a broad spectrum of cell-intrinsic and cell-extrinsic processes (e.g., host immune response) during cancer development and progression. Multiple studies have now demonstrated that GM-CSF can be anti-tumorigenic or pro-tumorigenic. The production of GM-CSF by tumor cells was found to be associated with the favorable clinical prognosis in patients with colorectal cancer (51). Conversely, GM-CSF upregulation was found to be associated with increased aggressiveness of various tumor types including bladder cancer (52, 53), colorectal carcinoma (54), glioblastomas (55) and head and neck cancers (56). Although a Causal link between the secretion of GM-CSF by tumor cells and the clinical outcome of specific cancer types is yet to be established, the plethora of literature suggests that GM-CSF behaves as a double-edged sword in cancer.

**Figure 2:**
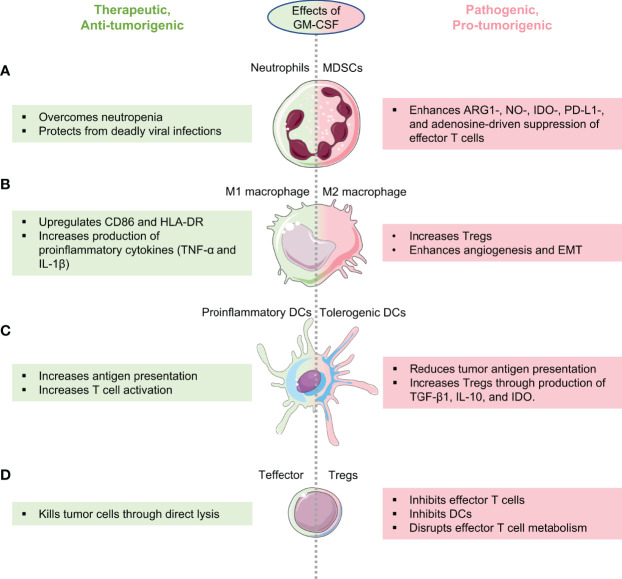
Therapeutic and pathogenic effects of GM-CSF on anti-cancer immune surveillance. Schematic highlighting how GM-CSF behaves as a double-edged sword in cancer by enhancing both anti- and pro-tumorigenic immune cells depending on its expression, cancer type, and tumor immune microenvironment. GM-CSF’s role in enhancing anti-tumor immune surveillance is shown in green and its role in reprogramming immune cells to the pro-tumorigenic phenotype is shown in pink. **(A)** GM-CSF enhances the production of neutrophils enabling patients with cancer to flight neutropenia (left), while also having the potential to convert neutrophils to pathogenic, cancer-promoting myeloid-derived suppressor cells (MDSCs, right). **(B)** Depending on the cancer, GM-CSF can reprogram macrophages to the tumor suppressive M1 phenotype (left) or to the tumor-promoting M2 phenotype (right). **(C)** GM-CSF can convert dendritic cells in cancers to the pro-inflammatory phenotype with better antigen-presentation capabilities to cytotoxic T cells (left) or to the tolerogenic phenotype that suppress cytotoxic T cells at the expense of regulatory cells (right). **(D)** By regulating myeloid cells that bridge innate and adaptive immune responses as outlined in **(A–C)**, GM-CSF can enhance the function of anti-cancer effector T cells (left) or induce regulatory T cells (right).

In-depth understanding of the anti- or pro-tumorigenic functions of GM-CSF in each cancer type and the causes underlying these functions is essential for rationally harnessing this cytokine for cancer treatment. Below, we outline the mechanisms underlying the therapeutic and pathogenic roles of GM-CSF in cancers (**Figure 2**). We describe strategies that are currently used in the clinic to harness the therapeutic benefits of GM-CSF while minimizing its pro-tumorigenic functions (**Table 1**). We then provide insights into the currently used and potential strategies to harness GM-CSF’s therapeutic potential against cancer while concurrently minimizing its deleterious effects. Finally, we discuss the future of GM-CSF-based therapies.

**Table 1:** GM-CSF formulations used to treat cancer patients in the clinic and in clinical development.

GM-CSF-Based Therapy	Type of cancer	Status of clinical development	Reference
Chemotherapy+ Leukine (sargramostim)	Acute Myelogenous Leukemia	FDA approved	
Leukine (sargramostim) after autologus and allogenic BMT	Non-Hodgkin’s lymphoma (NHL), acute lymphoblastic leukemia (ALL) and Hodgkin’s disease	FDA approved	
**Monotherapy**			
GM-CSF	Prostate Cancer	NCT00908141 NCT0027428	
GM-CSF	Ovarian Cancer Fallopian Tube Cancer	NCT00157573	
GM-CSF	Kidney Cancer Metastatic Cancer	NCT00006483	
**Combination with chemotherapy**			
GM-CSF+ G-CSF+ (Cyclophosphamide/Cyclophosphamide+ Etoposide)	Myeloma/Lymphoma	Clinical trial	(57)
GM-CSF + different Chemotherapy	Colon and Rectal Cancer	NCT00257322	(58)
GM-CSF + Docetaxel	Prostate Cancer	NCT00488982	
GM-CSF + Mitoxantrone	Prostatic Neoplasms	NCT00477087	
**Combination with monoclonal antibody**			
Leukine (sargramostim) + Herceptin	Breast Cancer	NCT00429104	
Leukine (sargramostim) + edrecolomab	Colorectal Cancer	NCT00002664	
GM-CSF + Nivolumab + Ipilimumab	Metastatic Cutaneous Melanoma	NCT02339571	
**Combination with cancer vaccines**			
PSA/IL-2/GM-CSF (complete vaccine)	Prostate Cancer	NCT02058680	
GVAX^/^ Vaccine (GM-CSF secreting prostate cancer vaccine)	Prostate Cancer	NCT00140374	
GM-CSF + Dendritic Cell/Tumor Fusion Vaccine	Ovarian Cancer Primary Peritoneal Cancer Fallopian Tube Cancer	NCT00799110	
Leukine (sargramostim) + NeuVax™ vaccine (E75 synthetic peptide combined with GM-CSF)	Breast Cancer with Low to Intermediate HER2 Expression	NCT01479244	(59)
GM-CSF + TroVax^/^ (vaccinia virus encoding the human oncofetal antigen 5T4)	Prostate Cancer	NCT00448409	
Leukine (sargramostim) + HER-2/neu peptide vaccine	Breast Cancer Lung Cancer Ovarian Cancer	NCT00003002	
Leukine (sargramostim) + iNeo Vac P01 (peptide vaccine)	Pancreatic Cancer Pancreatic Carcinoma	NCT03645148	(60–62)
GM-CSF + UV1 synthetic peptide vaccine	Non-small Cell Lung Cancer	NCT01789099	(63)
**Combination with cancer vaccines and monoclonal antibody**			
GM-CSF + UV1 vaccine + ipilimumab + nivolumab	Melanoma Lung cancer	NCT04382664 NCT04300244	
Leukine (sargramostim) + Galinpepimut-S (WT1 peptide vaccine) + Nivolumab	Mesothelioma Pleural Mesothelioma Wilms Tumor	NCT04040231	
Leukine (sargramostim) + pTVG-HP (plasmid DNA vaccine) + Nivolumab	Prostate Cancer	NCT03600350	
**GM-CSF neutralization**			
lenzilumab (anti GM-CSF)	Chronic Myelomonocytic Leukemia (CMML)	NCT02546284	
lenzilumab + Axicabtagene Ciloleucel + Cyclophosphamide + Fludarabine	Relapsed/Refractory Large B-cell Lymphoma	NCT04314843	
Lenzilumab + Selinexor + Interferon alfa	Cancer patients with Covid 19	NCT04534725	

### 2.1 Therapeutic Effects of GM-CSF in Cancer

#### 2.1.1 Immunostimulatory Effects of GM-CSF Contribute to Its Anti-Cancer Functions

The ability of GM-CSF to stimulate the production, maturation, and activation of neutrophils, macrophages, and DCs contributes largely to its anti-tumorigenic effects. Below, we detail the mechanisms by which GM-CSF mediates anti-cancer immune responses.

##### 2.1.1.1 GM-CSF Restores Neutrophil-Driven Immune Responses in Cancers

Of the downstream immune effectors of GM-CSF, neutrophils are particularly important because they are the most abundant WBCs and therefore, one of the first immune cell types to respond to infections (64) and cancers. Because cancer patients exhibit severe neutropenia after bone marrow transplantation, chemo- and other forms of therapy, they are at particularly high-risk of morbidity from infections than the healthy population (65–67). Administering GM-CSF to patients with cancer raises neutrophil numbers to levels required to protect the patient from contracting deadly infections including acquired immune deficiency syndrome (AIDS) (68–70). Because the anti-tumor potential of neutrophils has been recently appreciated in many cancers (71–74), we predict that reversal of neutropenia using GM-CSF may provide the added advantage of sustaining tumor regression in addition to protecting the patient with cancer from infections. However, this hypothesis requires testing.

##### 2.1.1.2 GM-CSF Activates Anti-tumorigenic Macrophages and DCs

GM-CSF promotes anti-tumor immune responses by activating monocytes/macrophages and enhancing DC differentiation (75). GM-CSF induces an inflammatory profile in human monocytes, which includes an upregulated expression of HLA-DR and CD86 molecules and increased production of TNF-α and IL-1β (75). GM-CSFR signaling also induces the polarization of tumor-associated macrophages to the anti-tumorigenic M1-like MHC-II^hi^ phenotype (76). GM-CSF-producing mouse and human colorectal tumors enhance local recruitment and activation of DCs that present tumor antigens to T cells in tumor-draining lymph nodes and activate other innate and adaptive host immune cells such as granulocytes, macrophages, and natural killer (NK) cells (77).

##### 2.1.1.3 GM-CSF Promotes Anti-Cancer T Cell Responses

Blockade of GM-CSF has been shown to impair the functionality of T cells (12) indirectly through the downmodulation of antigen presenting cells (APCs) including monocytes and DCs (14). GM-CSF blockade converts the GM-CSF-induced proinflammatory profile in monocytes characterized by the upregulation of HLA-DR and CD86 and production of TNF-α and IL-1β to an immunomodulatory one characterized by IL10 and CXCL-11 production, and suppression of T cell proliferation (36). After immunization with keyhole limpet hemocyanin (KLH), GM-CSF deficient mice exhibit impaired CD4^+^ T cell proliferation and IFNγ production and do not generate antigen-specific CD8^+^ T cells (14). GM-CSF was found to improve the anti-tumor effects of B16 melanoma tyrosinase-related protein 2 (TRP-2) peptide vaccines in combination with anti-CTLA-4 antibody by increasing the frequency of TRP-2-specific, IFN-secreting T cells in spleen and lymph nodes *via* DC priming and activation (78). GM-CSF alters the Th1/Th2 cytokine balance by enhancing the antigen-induced immune responses mediated by DCs (79–81). Bicistronic DNA vaccine co-expressing human immunodeficiency virus (HIV) gp120 and GM-CSF dramatically enhanced HIV specific CD4^+^T cell responses (82),thus, suggesting that GM-CSF may protect against virus-induced tumors. More recently, GM-CSF was shown to drive anti-tumor CD4^+^ and CD8^+^ T cell immune responses by activating IRF-5 in eosinophils in the tumor microenvironment (83).

#### 2.1.2 Direct Inhibitory Effect of GM-CSF on Tumor Cell Growth

Some tumors including colorectal, breast, and non-small cell lung cancers (NSCLC) aberrantly express GM-CSFR and secrete GM-CSF (77, 84, 85). In such GM-CSF- and GM-CSFR- expressing cancers, GM-CSF suppresses the proliferation of malignant cells by inducing arrest at the G0/G1 phase of the cell cycle and enhances their differentiation (84, 86, 87). Being an inducer of differentiation, GM-CSF was also found to eradicate cancer stem cells (CSC), the therapeutically resistant and most immature population in tumors. For example, treatment of small cell lung cancer cells with GM-CSF reduced proliferation of resistant clones in clonogenic assays and promoted their differentiation (86, 87). In another study, transducing GM-CSF into a CSC-derived from a murine breast cancer cell line reduced its colony forming ability *in vitro* and tumor formation potential *in vivo* (88).

#### 2.1.3 Inhibition of Angiogenesis by GM-CSF

Soluble vascular endothelial growth factor receptor 1 (sVEGFR-1) plays a regulatory role in VEGF-mediated angiogenesis by sequestering VEGF from VEGFR2, the main signaling receptor with tyrosine kinase activity. Eubank et al. showed that treatment of human monocytes with GM-CSF upregulated sVEGFR-1 thereby inhibiting angiogenesis and endothelial cell migration (89). Similarly, under hypoxic conditions, GM-CSF stimulates macrophages to secrete high levels of sVEGFR-1 and neutralize the activity of VEGF leading to reduced tumor growth, angiogenesis, and metastasis (90–92).

#### 2.1.4 Applications of GM-CSF in Cancer Treatment

##### 2.1.4.1 Direct Administration of GM-CSF

The therapeutic function of GM-CSF in cancer is best exemplified in patients with acute lymphoblastic leukemia (ALL), acute myeloid leukemia (AML), Hodgkin- and non-Hodgkin- lymphomas (HL/NHL), where recombinant human GM-CSF (Sargramostim or Leukine^®^) is used to stimulate early stem cells in donors prior to their harvesting for peripheral stem cell transplant and to stimulate recovery of HPCs after bone marrow transplantation (93).

In patients receiving chemotherapy for solid tumors, sargramostim (GM-CSF) has been shown to be beneficial as an immunostimulatory adjuvant to elicit antitumor immunity, and improve the overall condition of the patient (94). Subcutaneous administration of GM-CSF at a dose of 125 μg/m^2^ for 14 days in different cycles for a year has been shown to prolong survival by two years in patients with surgically resected stage III/IV melanoma in comparison to patients who did not receive GM-CSF (95, 96). Administration of low-dose GM-CSF in 19 patients with breast cancer, recurrent ovarian carcinoma, metastatic endometrial carcinoma, and recurrent squamous cell cancer of the cervix uteri with history of chemotherapy failure resulted in complete remission in 5% of patients and partial remission in 31.5% of patients (84).

##### 2.1.4.2 GM-CSF-Producing Oncolytic Viruses

Although intralesional injection of GM-CSF increases the numbers of and activates DC and T cells in the tumor microenvironment, its clinical usage has been limited because finding tumors which are accessible for injection and achieving a systemic immune response in these patients is difficult (97). Despite the challenges encountered during the intratumoral delivery of GM-CSF, oncolytic virus encoding GM-CSF when administered into the tumor showed promising results. Replication-defective Herpes Simplex Virus (HSV) expressing GM-CSF inhibited tumor growth in a Harding–Passey melanoma mouse model and improved survival of tumor-bearing mice (98). The herpes simplex virus constructed with the deletion of neurovirulence factors ICP34.5 and ICP47 and insertion of GM-CSF demonstrated anti-tumor effects *in vitro* and *in vivo* (99). The first GM-CSF-producing oncolytic virus immunotherapy approved by the Food and Drug Administration (FDA) was talimogene laherparepvec (T-VEC) for the treatment of unresectable stage IIIB/IV melanoma. T-VEC is a genetically modified live HSV-1 which decreases the infiltration of CD4^+^FoxP3^+^ Tregs and CD8^+^FoxP3^+^ suppressor T cells into the tumor microenvironment (100).

##### 2.1.4.3 GM-CSF-Based Anti-Cancer Vaccines

Apart from being used as a monotherapy, anti-tumor immune responses mediated by GM-CSF are heightened by combining it with anti-cancer vaccines. These include GM-CSF-secreting cancer cell vaccines, GM-CSF-fused tumor-associated antigen protein-based vaccines, and GM-CSF-based DNA vaccines. In a seminal study by Dranoff et al., irradiated autologous murine melanoma cell-based vaccines that were engineered to secrete GM-CSF had improved anti-tumor potential in comparison to unmodified melanoma cells that did not secrete GM-CSF (101). This heightened anti-cancer potential of GM-CSF-secreting melanoma cells was found to be mediated by the activation of host CD4^+^ and CD8^+^ T cells (101). In a clinical trial that followed, Dranoff and colleagues strengthened their murine findings in patients with metastatic melanomas by showing that GM-CSF-secreting melanoma cell-based vaccines improve clinical outcomes and mediate tumor shrinkage by activating cytotoxic T cell-mediated immune responses (102). Similarly, GM-CSF-secreting breast tumor cell vaccine showed antigen-specific CD8^+^ T-cell responses and improved overall survival (103). In metastatic hormone refractory prostate cancer (HRPC), allogeneic prostate cancer cell lines engineered to secrete GM-CSF (GVAX-PCa) were found to be well tolerated and improve anti-cancer immune responses (104). Recently, tumor antigen-loaded GM-CSF-producing myeloid cells derived from induced pluripotent stem cells (iPSCs) promoted CD8^+^ T cell homeostatic proliferation and T cell infiltration into the tumor tissue (105).

GM-CSF is used as an adjuvant therapy to activate cellular and humoral anti-tumor immune responses in the clinic. Sipucleucel-T (Provenge^/^) was the first FDA approved and only autologous DC loaded with prostate acid phosphatase antigen (PAP) and GM-CSF, which led to the recruitment of PAP-specific T cells into the prostate cancer microenvironment (106, 107). However, in patients with metastatic prostate cancer, using GM-CSF as an adjuvant in the DNA vaccine encoding the androgen receptor ligand-binding domain (pTVG-AR, MVI-118) had no additional therapeutic benefit than the vaccine alone (108). After administration as an adjuvant along with prostate cancer vaccine in patients from five different clinical trials, GM-CSF was found to increase antibodies against tumor-associated proteins (109). GM-CSF also increased immunoglobulin production, DC, CD4^+^, and CD8^+^ T cell counts, and tumor-specific lymphocyte cytotoxicity when administered together with the MB49 bladder cancer stem cell vaccine in a mouse model (110, 111). Addition of GM-CSF as an adjuvant to allogeneic melanoma cell-line vaccines was beneficial in raising robust, long-lasting anti-tumor immune responses (112–114). The vaccine combination of nelipepimut-S (NP-S) and GM-CSF was shown to be safe and increase immunity against herceptin (Her2) in patients with breast cancer (115). In a clinical trial of patients with refractory pancreatic cancer where GM-CSF was used as an adjuvant during administration of personalized neoantigen peptides (iNeo-Vac-P01), higher IFN-γ blood titer and increased counts of CD4^+^ and CD8^+^ effector memory T cells were observed after vaccination (60). Administration of the human telomerase reverse transcriptase (hTERT) vaccine UV1 along with GM-CSF as an adjuvant activated anti-cancer T cell responses in 67% of patients with NSCLC (63). Although GM-CSF was used as an adjuvant in vaccines to treat NSCLC and pancreatic cancer, the extent to which GM-CSF increases the efficacy of the cancer vaccines used in these studies remains unknown.

### 2.2 Pathogenic Effects of GM-CSF in Cancer

#### 2.2.1 Immunomodulatory Effects of GM-CSF Contribute to Its Pro-Tumorigenic Functions

A large body of experimental evidence suggests that GM-CSF supports tumor development. For example, aberrant expression of GM-CSF and its receptors has been found in many cancers such as glioblastoma, small cell carcinoma, skin carcinoma, meningiomas, colon cancer, head and neck cancer and lung cancer (77, 116, 117). GM-CSF promotes cancer progression by regulating the tumor microenvironment involving macrophages, myeloid-derived suppressor cells (MDSCs), promoting epithelial to mesenchymal transition (EMT), angiogenesis, expression of immune check point molecules, as detailed below.

##### 2.2.1.1 GM-CSF Promotes Generation of Tumor-Associated Macrophages and Myeloid Derived Suppressor Cells

Tumor-associated macrophages (TAMs) are key pro-tumorigenic immune cells in the cancer microenvironment. TAMs inhibit antitumor immune responses by secreting cytokines, chemokines, and growth factors that promote tumor growth and progression. Polarization of TAMs into the tumor-promoting M2 phenotype is controlled by factors secreted by the tumor and surrounding cells (118), such as C-C motif chemokine ligand 2 (CCL2) (119). CCL2 binds to its cognate receptor CCR2 to mediate its tumor promoting effect (120, 121). Despite being predominantly an M1-inducing factor (122), GM-CSF can induce the production of CCL2 from T cells in the tumor microenvironment and the expression of CCR2 on macrophages thus polarizing them to the metastasis-promoting the M2 phenotype (120, 121). In Epstein–Barr virus (EBV) associated nasopharyngeal carcinoma (NPC), cancer cells differentiate monocytes to a TAM-like phenotype by secreting GM-CSF in an NK-κB-dependent manner. TAMs then secrete CCL-18 leading to EMT. Concordant with these findings, neutralization of GM-CSF was significantly found to reduce NPC metastasis (123). In pancreatic ductal adenocarcinoma (PDAC), high expression of GM-CSF and HIF1α is associated with perineural invasion (PNI) and poor clinical outcomes in patients (124). The tobacco carcinogen, nitrosamine 4-(methyl nitrosamino)-1-(3-pyridyl)-1-butanone (NNK), is known to potentiate PDAC development *via* GM-CSF-mediated activation of cyclic AMP response element-binding protein (CREB). CREB inhibition was found to block GM-CSF-induced recruitment of TAMs and regulatory T cell (Treg) expansion (125). In another PDAC study, Waghray et al. found that GM-CSF secreted from cancer-associated mesenchymal stem cells (CA-MSCs) drives PDAC cell proliferation and metastasis (126).

MDSCs are heterogeneous cells of myeloid origin that resemble activated monocytes and neutrophils with immunosuppressive functions (127). GM-CSF differentiates CD11b−Gr1− bone marrow progenitor cells into MDSCs (128). As discussed earlier in this review, depending on the GM-CSF’s signaling strength, which is a function of its concentration, it can activate PI3K/Akt (low GM-CSF) and JAK2/STAT5 (high GM-CSF) pathways (24, 25, 129). The signaling events downstream of PI3K, such as, Akt isoform 2 (Akt2) activation (130, 131), and downregulation of Src homology 2-domain-containing inositol-5′-phosphatase (SHIP) (132) are known to impart an immunosuppressive phenotype to myeloid cells. Loss of the negative regulator of PI3K, phosphatase and tensin homolog (PTEN), therefore, leads to an immunosuppressive tumor microenvironment by upregulating MDSC, Tregs, and TAM (133). Mammalian target of rapamycin (mTOR) has also been implicated in GM-CSF- driven MDSCs development. The PI3K/Akt/mTOR axis stabilizes the expression of iNOS in mice or indoleamine 2,3-dioxygenase (IDO) in human monocytes that leads to their immunosuppressive functions (134). GM-CSF-mediated activation of the JAK2-STAT5 pathway also promotes MDSC development by downregulating the transcription of IRF8 (135). In an independent study, deficiency of important negative regulators of the JAK-STAT signaling pathway, the cytokine-inducible SH2-containing protein (CIS) family members (136), biased macrophages towards an immunosuppressive M2-like phenotype with reduced IL-12 production. Such skewing of the CISH−/− macrophages to the pro-tumorigenic M2 phenotype was found to result from their reduced IRF-8 expression (137). In addition to IRF-8, GM-CSF-mediated STAT5 activation in MDSCs causes overexpression of fatty acid transporter protein 2 (FATP2), and this in turn controls uptake of arachidonic acid (AA) and synthesis of prostaglandin E2 (PGE2) by MDSCs. Concordantly, deletion or pharmacological inhibition of FATP2 abrogated immunosuppressive functions of MDSC and delayed tumor regression (138).

GM-CSF-driven recruitment of MDSC in tumor microenvironment (139) inhibits adaptive anti-tumor immune responses *via* multiple mechanisms outlined below. GM-CSF enhances IL-4RA expression on MDSCs (140). In response to IL-4, MDSCs secrete arginase 1 (ARG1) (141) that depletes arginine in the tumor microenvironment. Because arginine is an important factor for the proliferation and metabolic fitness of T cells (142), its depletion by ARG1 causes the downregulation of CD3 ζ-chain thus impairing T cell activation and function (143). Increased arginase activity has been associated with poor prognosis in cancers including in AML (144), head and neck squamous cell carcinoma (145), pancreatic cancer (146), ovarian cancer (147), colorectal cancer (148), and in hepatocellular carcinoma (HCC) (149). In addition to ARG1, GM-CSF-induced CD11b^+^Gr1^+^ MDSCs inhibit functions of effector T cells *via* the nitric oxide synthase (iNOS) pathway that produces nitric oxide (NO) (150). NO inhibits tyrosine phosphorylation of JAK3 and STAT5 and other intracellular signaling proteins such as ERK and AKT thus suppressing antigen-stimulated T cell proliferation in response to IL-2 (151, 152). In some cancers, ARG1 and iNOS together inhibit T cell effector functions (141). In PDAC, MDSCs that develop from tumor-derived GM-CSF also inhibit antigen-specific CD8^+^ T cell responses (153). Additionally, it was found that genetic ablation of CD73, a surface ecto-5’-nucleotidase that converts adenosine monophosphate (AMP) to immunosuppressive adenosine, lowers GM-CSF in the tumor microenvironment thereby preventing MDSC development and improving CD4^+^ and CD8^+^ effector T cell responses (154). In hepatic cancers, liver MDSCs co-express GM-CSFR, PD-L1 and IDO and show higher STAT-3 activation (139). PD-L1 and IDO are potent inhibitors of T-cell effector functions (155). Blocking GM-CSF or its receptor diminished IDO and PD-L1 expression in liver MDSCs and restored intrahepatic antitumor immunity (139). GM-CSF-driven upregulation of prostaglandin E2 (PGE2) expression in MDSCs (138) can potentially suppress effector functions of macrophages, cytotoxic T and NK cells and promote pro-tumor Th2, Th17, and Treg responses (156).

##### 2.2.1.2 Regulation of Immune Checkpoints by GM-CSF

In gastric cancer, tumor-derived GM-CSF stimulates neutrophils to express PD-L1. These GM-CSF-activated neutrophils suppress T cell proliferation and IFN-γ production *in vitro* and support the development of gastric cancer *in vivo* in a PD-L1-dependent manner (157). Similarly, in extranodal natural killer/T cell lymphoma (ENKTL) patients, treatment with GM-CSF suppresses the antitumor immune response by upregulating the PD-L1 expression through JAK2/STAT5 pathway (158).

#### 2.2.2 GM-CSF Drives Epithelial to Mesenchymal Transition

EMT is mediated by related zinc finger proteins such as Snail, Slug, Twist-related protein 1 (TWIST1), zinc-finger E-box-binding homeobox 1 (ZEB1), and ZEB2 (159). In breast cancer cells, these EMT-promoting transcription factors are known to induce the production of GM-CSF and other inflammatory cytokines such as IL-6, IL-8, soluble intercellular adhesion molecule 1 (ICAM1), and plasminogen activator inhibitor 1 (PAI1) (160, 161) and enhance cancer progression. In breast cancer, tumor cells secrete high levels of GM-CSF that activate TAMs to induce EMT through CCL18 secretion (162). CCL18 in turn increases the expression of EMT promoting transcription factors in a positive feedback loop to further promote migration and invasion of cancer cells to distant sites (163). Therefore, CCL18 represents a promising therapeutic target for blocking GM-CSF-induced cancer metastasis (162). In colon cancer, although GM-CSF secreted by cancer cells is initially anti-tumorigenic (51), chronic exposure to GM-CSF leads to EMT because of the activation of MAPK/ERK and ZEB1 pathways. Such colon cancer cells stimulated by GM-CSF over a long term show higher migratory capacity *in vitro* and *in vivo* as well as resistance to chemotherapy (164). GM-CSF was found to induce EMT of PDAC cell lines by downregulating E-cadherin and upregulating the expression of TWIST1 and vimentin through activation of STAT3 signaling (126).

#### 2.2.3 Promotion of Angiogenesis by GM-CSF

Angiogenesis drives the metastasis of cancer and is mediated by factors such as VEGF, fibroblast growth factor-2 (FGF-2), and platelet-derived growth factor (PDGF) (165). As with its immunomodulatory functions, GM-CSF can suppress or stimulate angiogenesis in cancer in a context dependent manner. In fact, inducing blood vessel growth (166) as well as improving vascularization during wound healing and tissue regeneration (167) is a normal physiological function of GM-CSF. The endothelial angiopoietin (Ang)–Tie growth factor receptor pathway regulates vascular remodeling during inflammation and cancer metastasis (168). GM-CSF regulates early and later stages of blood vessel formation by modulating the expression of VEGF, expression ratio of Ang-1/Ang-2, and the phosphorylation of Tie-2 (169). Also, GM-CSF mediate enhanced proliferation of endothelial progenitor cells (EPCs) during neovascularization by upregulating cyclins D1 and E *via* PI3K and MAPK signaling pathways (170). Apart from its direct proangiogenic effect on EPCs, GM-CSF can also enhance angiogenesis indirectly by promoting the migration and recruitment of proangiogenic granulocytes in certain types of tumors (171).

GM-CSF has also been implicated in Toll-like receptor 2/6 (TLR2/6)-induced angiogenesis, wherein, induction of GM-CSF in endothelial cells by TLR2/6 agonist macrophage-activating lipopeptide of 2 kDa (MALP-2) promotes their proliferation and migration (172). Therefore, it is not surprising that GM-CSF plays a role in driving angiogenesis in cancer.

In colitis-associated cancer (CAC), colonic epithelial cells (CEC) secrete VEGF in response to commensal microbiota-derived LPS that induce GM-CSF expression in CEC. Neutralizing GM-CSF *in vivo* significantly reduced the VEGF release from CEC and abrogated CAC development (173). In head and neck squamous cell carcinoma (HNSCC), secretion of GM-CSF together with VEGF and PDGF by HNSCC cells correlated with increased micro vessel density and poor clinical prognosis (56). In preclinical studies using models of metastatic breast cancer, the Axl kinase was found to promote angiogenesis by stimulating secretion of GM-CSF from the tumor cells (174). Secretion of GM-CSF by progenitor cells in white adipose tissue was found to stimulate angiogenesis and breast cancer progression (175). In gastric cancer, overexpression of the cytidine monophospho-N-acetylneuraminic acid hydroxylase pseudogene (CMAHP) drove angiogenesis by inducing the transcription of GM-CSF (176).

#### 2.2.4 Overcoming the Pro-Tumorigenic Functions of GM-CSF During Cancer Treatment

The above-described pro-tumorigenic properties of GM-CSF can be reversed either by directly targeting GM-CSF and its receptor in cancers or by targeting downstream effectors of GM-CSF signaling. We briefly detail some of these strategies that have been explored in preclinical and clinical studies of cancer.

One of the first examples of targeting the GM-CSFR directly on cancer cells is the combination of recombinant fusion toxins with diphtheria toxin or pseudomonas endotoxin to treat myeloid malignancies and gastrointestinal cancers (177–179). *In vitro* studies using mavrilimumab, a human monoclonal antibody targeting GM-CSFRα, have shown to prevent the polarization of human monocytes into PD-L1-expressing MDSCs and to restore T cell proliferation (180). Another strategy to block GM-CSF signaling has been the use of neutralizing antibodies against GM-CSF. For example, neutralization of GM-CSF prevented MDSC accumulation and abrogated HCC progression (181). More recently, the αGM-CSF antibody was found to reprogram the tumor microenvironment from immunosuppressive to anti-tumorigenic in intrahepatic cholangiocarcinoma by reducing the expression of ARG1 and PD-L1 in granulocytes and monocytes, inhibiting M2 TAMs, and augmenting activation and tumor-infiltration of CD8^+^ T cells, leading to a pan-myeloid repolarization towards the pro-immunogenic, anti-tumor phenotype (182). Similar paradigms of restoration of tumor immunosurveillance using anti-GM-CSF antibodies to neutralize GM-CSF secreted by breast cancer cells have been demonstrated by Su et al. (183). Finally, as a surrogate to neutralizing GM-CSF or targeting its receptor, other strategies that target downstream effectors of GM-CSF signaling have also been explored. These include, targeting the Axl kinase (174) and treatment with agents such as metformin that mimic agents that neutralize GM-CSF (175) to block angiogenesis and EMT, as well as the pharmacological inhibition of proteins such as FATP2 to prevent GM-CSF-driven PMN generation (138).

## 3 Future of GM-CSF-Based Anti-Cancer Therapies: Challenges and Solutions

Prospects of GM-CSF-based treatments depend on the identification of underlying mechanisms that regulate its pleotropic pro- and anti-tumorigenic functions in the pathogenesis of each cancer. This is because, whether GM-CSF promotes tumor progression or regression is dependent on the cancer type and unique tumor microenvironments, as well as the dose, duration, and frequency of GM-CSF administration. Below, we detail what is known and what is yet to be uncovered for rationally harnessing the therapeutic potential of GM-CSF in cancers. Finally, we provide insights into current and future technological approaches that will enable researchers to clarify the function of GM-CSF in treatment of hematological malignancies and solid tumors.

### 3.1 Optimizing Dosage and Route of Administration of GM-CSF Based Therapies

A critical factor that decides whether GM-CSF-based therapies exert anti- or pro-tumorigenic effects is its dose (24, 117, 150). In preclinical studies, it was shown that GM-CSF differentiated the granulocyte-macrophage progenitor cells to macrophage or granulocyte colonies in a concentration-dependent manner (184). High doses of GM-CSF correlate with cell survival and proliferation while low concentrations of GM-CSF support cell viability but do not promote cell proliferation (24, 185).

A rationale underlying the development of GM-CSF-based vaccines is the ability of GM-CSF to promote DC maturation and thereby enhance the presentation of tumor antigens. This benefit of GM-CSF is countered by its ability to induce tumor-promoting MDSCs. Of note, this immunosuppressive effect of GM-CSF is seen only during its systemic administration but not when GM-CSF secretion is localized to the tumor microenvironment (150). Hence, targeted delivery of GM-CSF to the cancer site may be more beneficial than its systemic administration. That said, because prolonged targeting of GM-CSF based therapies to the tumor microenvironment will eventually lead to the development of tolerogenic myeloid cells, dosage of GM-CSF to be administered must be carefully determined. Such determination of dosage will be made considering the differences between cancer types and the complex heterogeneity of the tumor microenvironment. One potential strategy to alleviate dosage dependent side effects of GM-CSF is to engineer GM-CSF-producing APCs. These engineered APCs might prevent the systemic side effects of GM-CSF which is usually seen during intravenous administration of GM-CSF. This combination of GM-CSF with APCs have shown improved antitumor cytotoxic T cell-mediated response (105). Other potential strategy is fusing GM-CSF with Fc portion of target specific monoclonal antibody (mAb), these fusion proteins are also known as immunokines. These immunokines have localized effects and reduced toxicity contrary to systemic administration of therapeutic cytokines. Cytokine mutagenesis or protein engineering techniques can also be employed to modulate GM-CSF stability, specificity, half-life and activity to optimize its functions in a context dependent manner (186).

### 3.2 Rational Combinatorial Regimens With GM-CSF

At the outset, many patients with hematological malignancies and solid tumors require GM-CSF to shorten the duration of neutropenia caused by administration of immune system-ablating chemo- and radiation therapies (68–70). However, chronic exposure to GM-CSF in such patients can eventually lead to the manifestation of its deleterious pro-tumorigenic effects including MDSC generation, immune exhaustion, and tumor metastasis. This problem can be addressed in multiple ways. First, it is crucial to examine whether GM-CSF-driven restoration of neutropenia improves the clinical prognosis in a specific treatment setting. For example, in leukemia, neutrophil recovery after GM-CSF administration is marked only in patients who received autologous bone marrow transplants but not in patients who received allogeneic transplants. Furthermore, in the allogeneic transplant setting, GM-CSF and G-CSF do not impact graft vs host disease or overall survival. Therefore, it is recommended that GM-CSF and G-CSF be only used in patients with leukemia who face a delay in the reconstitution of the allogenic bone marrow transplant (68).

For patients in whom GM-CSF initially restores anti-infection and anti-tumor immune surveillance and improves quality of life, but leads to pro-tumorigenic effects over the long-term, the following approaches may be used. To prevent the development of immunosuppressive MDSCs during chronic treatment with GM-CSF, combining GM-CSF with agents that block specific isoforms of PI3K such as phosphatidylinositol 3-kinase (PI3K) δ and PI3Kγ which are expressed in the hematopoietic lineage (187), Akt (131, 188) or mTOR (e.g., rapamycin, everolimus) (134) are potential strategies. Other pharmacological agents that block MDSC production including FATP2 inhibitor (189), the STAT3-specific inhibitor static (190), and the receptor tyrosine kinase (RTK) inhibitor sunitinib (189) can be combined with GM-CSF treatment to avoid potential immunosuppressive side effects of long-term GM-CSF treatment. Because GM-CSF suppresses the generation of beneficial M1 macrophages (132) that can produce the anti-cancer cytokine IL-12 in certain tumor types (118), IL-12-based therapies (191, 192) may represent another promising approach to overcome GM-CSF-mediated activation of suppressive myeloid cells.

Another approach harnesses GM-CSF’s ability to induce immune checkpoints in cancer by combining it with immune checkpoint inhibitors. Because GM-CSF induces PD-L1 expression in a JAK2/STAT5 dependent manner, treating cancers with dysregulated JAK/STAT5 signaling using GM-CSF would lead to disease progression. Mutation of *STAT5A* is frequently observed in cancers and GM-CSF is known to aggravate the disease through induction of PD-L1 expression. For example, it was observed that ENKTL cells with mutated *STAT5A* expressed higher levels of PD-L1 upon treatment with GM-CSF as compared to their wildtype counterparts (158). In NSCLC, higher expression of JAK-2 is correlated with increased PD-L1 gene expression and poor overall survival of patients (193). Therefore, if GM-CSF must be administered to the patient, then approaches that combine GM-CSF with checkpoint inhibitors would be beneficial.

In fact, in preclinical studies using melanoma and colon cancer animal models, tumor cell vaccines expressing GM-CSF and anti-PD1 exhibited profound antitumor T cell responses and improved the overall survival of tumor-bearing mice as compared to mice treated with either GM-CSF or anti-PD-1 alone (194, 195). In another melanoma study, blockade of PD1/PD-L1 unleashed the anti-tumor potential of GM-CSF-secreting group 2 innate lymphoid cells (ILC2s) (196). Similarly, combining GM-CSF with CTLA-4 blocking mAb ipilimumab improved tumor antigen-specific T cell responses in prostate (197) and pancreatic cancer (198) in comparison to either GM-CSF or ipilimumab alone. Therefore, combining GM-CSF with immune checkpoint inhibitors can convert a pro-tumorigenic immune microenvironment to an anti-tumorigenic one by enhancing the tumor antigen-specific adaptive immune responses.

Although the above combinatorial strategies are attractive to harness the beneficial effects of GM-CSF and concurrently suppress its pro-tumorigenic functions, additional studies are needed to determine which cancer types will benefit from these combination regimens and whether GM-CSF can be safely co-administered with other therapies or must be delivered in a staggered fashion.

### 3.3 Strategies to Overcome GM-CSF-Driven Cytokine Release Syndrome and Neurotoxicity

GM-CSF is the central mediator of cytokine release syndrome (CRS) and neurotoxicity in patients who have been administered CAR T cell-based therapies (199). CRS is characterized by a dramatic rise in the serum levels of inflammatory mediators such as C-reactive protein (CRP), cytokines (IL-6, IFN-γ, IL-10, IL-15, GM-CSF), chemokines (IL-8, monocyte chemoattractant protein-1 (MCP-1), macrophage inflammatory protein (MIP) 1α and 1β, CXC chemokine ligand 9 (CXCL9), CXCL10), and soluble cytokine receptors (sIL-2RA, sIL-6R, sgp130) (200–202). Tumor recognition by CAR-T cells leads to their massive proliferation and release of cytokines including GM-CSF (203). It has been postulated that the CAR-T cell-infused patients with an early increase in GM-CSF levels have higher propensity to develop severe CRS (204). An abnormal increase in GM-CSF leads to the expansion of myeloid cells (205). Myeloid cells then produce IL-6 (206), the most elevated cytokine in CAR-T-induced CRS (207). Tocilizumab, an IL-6R antagonist, that blocks IL-6-induced inflammatory pathways, is the only current treatment available to tackle CRS in the clinic. Of note, because IL-6 is not produced by CAR-T cells and is not essential for CAR-T cell functions, tocilizumab is a safe strategy to overcome GM-CSF-driven CRS (206). Alternate treatments to tocilizumab for reducing CRS and neuroinflammation are being investigated in preclinical studies; these include the neutralization of GM-CSF with lenzilumab (an anti-GM-CSF monoclonal antibody) (199) and GM-CSF−/− CAR T-cells (203).

Apart from exacerbating CRS, GM-CSF enhances neurotoxicity of CAR-T therapies by promoting the migration of inflammatory phagocytes to the central nervous system (CNS). These GM-CSF-stimulated myeloid-derived phagocytes produce reactive oxygen species leading to CNS inflammation and neurological defects. Increased expression of neuroinflammatory cytokines such as IL-1β, IL-6, and TNF-α in CNS have been correlated with dysregulated GM-CSF production (205, 208). Currently, there is no approved treatment available for CAR-T cell-induced neurotoxicity. Neutralization of GM-CSF with lenzilumab in patient-derived xenograft mouse model of B-ALL treated with CAR-T cells alleviates neuroinflammation and symptoms of CRS by reducing migration of myeloid and T-cell migration to the CNS (199). Because GM-CSF ablation in CAR-T cells does not affect their anti-tumor effector functions and proliferation potential, GM-CSF−/− CAR-T cells may be a potential strategy to reduce CRS and neuroinflammation and eliminate the need to administer anti-CRS agents in patients undergoing CAR-T cell-based therapy for cancer (203).

## 4 Concluding Remarks

To summarize, GM-CSF exerts both pro- and anti-tumorigenic effects by modulating the global immune responses in cancer (**Figure 2**). Therefore, enhancing GM-CSF’s anti-cancer functions while concurrently minimizing its deleterious functions requires an understanding of how the tumor immune microenvironment is uniquely modulated by GM-CSF in specific cancer types. The advent of high-dimensional single-cell immune profiling approaches including cytometry by time of flight (CyTOF) (209), multiplexed ion beam imaging by the time of flight (MIBI-TOF) (210), CO-Detection by indEXing (CODEX) (211), and single cell RNA sequencing (scRNAseq) (211), allow one to determine the numbers, phenotype, activation states, and spatial distribution of multiple immune subsets in tumor, peripheral healthy tissue, and peripheral blood samples obtained from patients receiving GM-CSF-based therapies. These can be coupled with studies in mouse models of cancer where GM-CSF signaling can be modulated either through direct administration of GM-CSF-based therapies (**Table 1**) or through genetic means. Therefore, the current and ongoing technological and scientific advances in understanding the effects of GM-CSF at a single-cell, spatial, and temporal level in cancers will provide important insights into efficiently harnessing GM-CSF for treating cancers.

## Author Contributions

AK, ATK, and ASO wrote the manuscript. SS conceived and developed the scientific premise, supervised AK, ATK, and ASO wrote, and edited the manuscript. All authors contributed to the article and approved the submitted version.

## Funding

This work is supported by the following grants to SS: Translational Research Program Award from the Leukemia and Lymphoma Society (LLS 6624-21), Scholar Award from the American Society of Hematology, a Childhood Cancer Research Grant from the B+ Foundation, the P50 CA107399-12 National Cancer Institute Lymphoma SPORE Career Enhancement Program Pilot Award from the National Institutes of Health (PIs: Stephen J. Forman, Larry W. Kwak), and Research Start-Up from the Beckman Research Institute of City of Hope. The above funding sources together cover open access publication fees.

## Conflict of Interest

The authors declare that the research was conducted in the absence of any commercial or financial relationships that could be construed as a potential conflict of interest.

## Publisher’s Note

All claims expressed in this article are solely those of the authors and do not necessarily represent those of their affiliated organizations, or those of the publisher, the editors and the reviewers. Any product that may be evaluated in this article, or claim that may be made by its manufacturer, is not guaranteed or endorsed by the publisher.

## Glossary

**Table:**

GM-CSF	Granulocyte-macrophage colony-stimulating factor
PMNs	polymorphonuclear neutrophils
DCs	dendritic cells
TLR	toll-like receptor
IL	interleukin
Jak	Janus Kinase
pDCs	plasmacytoid DCs
HPCs	hematopoietic progenitor cells
CMP	common myeloid progenitor
APCs	antigen presenting cells
MDPs	macrophage/DC progenitors
AIDS	acquired immune deficiency syndrome
HLA	human leukocyte antigen
CD	cluster of differentiation
TNF	tumor necrosis factor
MHC	major histocompatibility antigen
NK	natural killer cells
VEGF	vascular endothelial growth factor
VEGFR1	vascular endothelial growth factor receptor 1
ALL	acute lymphoblastic leukemia
AML	acute myeloid leukemia
Hodgkin- and non-Hodgkin- lymphomas	HL/NHL
WBC	white blood cells
HSV	Herpes Simplex Virus
FDA	Food and Drug Administration
T-VEC	talimogene laherparepvec
MDSCs	Myeloid-derived suppressor cells
EMT	epithelial to mesenchymal transition
TAMs	tumor-associated macrophages
PAP	prostate acid phosphatase
CCL2	CC motif chemokine ligand 2
EBV	Epstein&ndash;Barr virus
NPC	nasopharyngeal carcinoma
PDAC	pancreatic ductal adenocarcinoma
PNI	perineural invasion (PNI)
NNK	nitrosamine 4-(methyl nitrosamino)-1-(3-pyridyl)-1-butanone
CREB	cyclic AMP response element-binding protein
Treg	regulatory T cell
CA-MSCs	cancer-associated mesenchymal stem cells
ARG1	arginase 1
iNOS	nitric oxide synthase
Jak3	Janus kinase 3 (Jak3)
STAT5	signal transducer and activator of transcription 5
AMP	adenosine monophosphate
IDO	indoleamine 2
3-dioxygenase (IDO)	
ENKTL	extranodal natural killer/T cell lymphoma
TWIST	Twist-related protein 1 (TWIST1)
ZEB	zinc-finger E-box-binding homeobox
FGF2	fibroblast growth factor-2
PDGF	platelet-derived growth factor
CAC	colitis-associated cancer
CEC	colonic epithelial cells
HNSCC	head and neck squamous cell carcinoma
PI3K	phosphatidylinositol 3-kinase
MAPK	mitogen-activated protein kinases
PD-1	programmed cell death 1
CRS	cytokine release syndrome
CNS	central nervous system
iCCA	intrahepatic cholangiocarcinoma
TRP-2	tyrosinase-related protein 2
CSC	cancer stem cells
Tg	transgenic
Treg	regulatory T cells
IRF4	interferon regulatory factor 4
sVEGFR-1	soluble form of membrane bound VEGF receptor-1
CIS	cytokine-inducible SH2-containing protein
ERK	extracellular signal-regulated kinases
CyTOF	cytometry by time of flight
MIBI-TOF	multiplexed ion beam imaging by the time of flight
scRNAseq	single cell RNA sequencing
CODEX	CO-Detection by indexing
Her2	Herceptin 2
hTERT	human telomerase reverse transcriptase
PAI1	plasminogen activator inhibitor 1
ICAM1	intercellular adhesion molecule 1
MCP-1	monocyte chemoattractant protein-1
MIP	macrophage inflammatory protein
CXCL	CXC chemokine ligand
PTEN	phosphatase and tensin homolog
SHIP	Src homology 2-domain-containing inositol-5&prime-phosphatase
PGE2	prostaglandin E2
CMAHP	cytidine monophospho-N-acetylneuraminic acid hydroxylase pseudogene (CMAHP)
HCC	hepatocellular carcinoma
EPCs	endothelial progenitor cells
mTOR	mammalian target of rapamycin
NSCLC	non-small cell lung cancer
FATP2	fatty acid transporter protein 2
Ang	angiopoietin
MALP-2	macrophage-activating lipopeptide of 2 kDa.
